# Minding the moving self: the centrality of body movement in the neurodynamics of the self and psychotherapeutic implications

**DOI:** 10.3389/fpsyt.2025.1726099

**Published:** 2025-12-18

**Authors:** Sharon Vaisvaser

**Affiliations:** School of Society and the Arts, Faculty of Humanities and Social Sciences, Ono Academic College, Kiryat Ono, Israel

**Keywords:** multidimensional self, predictive processing, body awareness, mirroring, embodied mentalization, interpersonal synchronization

## Abstract

Neuroscientific explorations of the self acknowledge the central role of the body and dynamic sensory-motor interactions in sense of self and mental functioning. The multidimensional self-concept comprises pre-reflexive bodily dimensions scaffolding higher-order mental self-representations, which relate to and rely on movement-based relational foundations. Disruptions in ongoing self-constitution, development and expansion call for psychotherapeutic work that recognizes and utilizes dynamic bodily aspects, and formulation of personalized treatment plans. This manuscript discusses the brain-body-mind interface, encompassing experiential and temporal-spatial dynamics supported by integrated hierarchical neural processes, in line with predictive processing accounts. Epistemic affordances, or action possibilities, are anchored in neural mechanisms and interlaced with psychotherapeutic work facilitating the generation of predictive models of the body in the world. Movement within the peripersonal space is linked to self-modeling, along with insights into interoceptive awareness covering multimodal sensory-motor integration that facilitates emotional and cognitive processing. Bodily-anchored, and movement-based temporal aspects of the self are discussed in terms of the ‘temporal thickness’ of experience and further elaborated in relation to mental time travel and autobiographical memory. This grounds the analysis of the implicit and explicit movement within therapeutic relationships. Intersubjective neural mechanisms of mirror-simulation and synchronization are shown to be associated with kinesthetic empathy, embodied mentalization and communicative means of mirroring, attuning, and synchronizing movement. By bridging neuroscientific and clinical perspectives on the embodied, multifaceted dynamics across the nested dimensions of the self, this manuscript outlines pathways for transformative interventions that foster neuroplastic movement toward self-integration.

## 1 Introduction

Mental health challenges are now widely seen as encompassing complex alterations of self-experience that emerge from the dynamic interactions among behavioral, cognitive, affective, and social processes that place the lived body at the core of these dynamics. Neuroscience research has progressively established the central role of the body in the experience and sense of self (1). Current conceptualizations of brain function as embodied, enactive and relational (2) recognize the dynamic nexus it forms with the body which significantly influences emotions, cognition, and overall mental health (3). The “mind”, which cannot be separated from the living body, manifests and integrates the current state of the entire organism as a bodily subject during its sensory-motor interactions with the (social) environment (4). Thus, in the ongoing experience of the self, perception, cognition and emotion find themselves in a mutual embrace with action (1, 5, 6). These approaches also support neurodiversity, by embracing the inherent interconnectedness of the brain, body, and environment, thus creating more inclusive and supportive environments (7).

The idea that perceptual, affective and cognitive processes are influenced by actual experiences has received substantial support from the well-established Bayesian Predictive Processing (PP) framework of brain function. Based on the Free Energy Principle as applied to any biological system that resists a tendency towards disorder, the PP framework describes how the brain actively (and enactively) infers latent or hidden causes of internal and external sensations in order to anticipate them formulate a reaction and minimize uncertainty (8). The dependence on embodied priors mostly occurs implicitly, without the need to explicitly remember past events (9). The brain is therefore perpetually engaged in a continuous crosstalk between lower and higher levels of the cortical hierarchy, which is consistent with the hierarchical signal exchange between cortical layers (10). Top-down predictions are integrated with bottom-up somatosensorial information, leading to either matches or mismatches, i.e., prediction errors (surprises), where salient prediction errors (having high precision) refine predictive models and drive action (11). This active engagement helps minimize prediction errors through behavior and interaction, thus further refining the brain’s predictive accuracy (12).

The embodied-embedded self, as a self-organizing system, needs continuous and identifiable boundary conditions, which are known as “Markov blankets”; i.e., the set of states that separates internal parts from external ones (13). “Markov blankets” act as a protective screen through which the individual, as a living being, can recognize and distinguish its internal states from the states of the external environment by inferring the internal or external causes of sensations and perceptions (14). Accordingly, selfhood is an agent’s embodied model in the world that constantly searches for evidence of its own existence through its actual activities (15), which give rise to more complex self-representations. Models (predictions) of the self in the world are generative and hierarchical; in other words, higher levels contextualize lower levels, and lower levels provide evidence for higher levels (16). Free energy minimization corresponds to maximizing self-model evidence, which implies the notion of “self-evidencing”; i.e., a free energy minimizing agent that will always try to maximize evidence for its own existence (14, 15). Selfhood, the self-organizing existence within a world separated from the self, relies on dynamic inferential processes, that are achieved through acting upon the world and sampling new sensory states, thus further conforming to goal or object-directed notions of affective intentionality (17, 18).

The critical role of the body and nonverbal behavior and interaction in psychotherapy have attracted increasing interest (19, 20). The 4E cognition principles (embodied, embedded, enactive, and extended) emphasize the multi-level factors of the patient and therapist interaction, including physiological and behavioral/movement parameters (21). A deeper understanding of the multifaceted body may enable psychotherapists to recognize and utilize a larger range of diverse therapeutic approaches and hence select and apply forms of therapy that are more optimally tailored and effective for the unique person in therapy.

Personalized psychotherapeutic care increasingly integrates embodied approaches that consider body movement as a core dimension of self-regulation, affective expression, and interpersonal connection, thus reflecting the growing neuroscientific evidence on the dynamic brain-body coupling in mental health and therapeutic change (18, 22, 23). Understanding mental health through the lens of brain-body coupling supports interventions that work *with* the patient’s embodied tendencies that are revealed in the shape and quality of movement, emerging within the therapeutic relationship. Dance/Movement Therapy (DMT) is a psychotherapeutic creative arts modality rooted in the idea of a body-mind nexus (24). DMT’s basic premise is that body movement both reflects and impacts inner emotional and mental states. Thus, the therapeutic use of movement in a relational constellation enables the patient to create a more embodied and coherent self-concept and narrative that fosters vitality and expressivity. Acknowledging and inviting expressions from the lived body hold the promise of enhancing self-connectedness, connections with others and with the world around (25, 26). Growing empirical evidence highlights the importance of the arts in the prevention, management and treatment of diverse disorders (27). DMT has been shown to be an effective intervention for a range of psychiatric, neurodevelopmental and neurodegenerative conditions that reduces symptoms while increasing quality of life and well-being, interpersonal skills, physical, emotional and cognitive functions (28–32).

Recognizing and analyzing the central role of the body and movement is directly relevant to DMT and offers broader contributions to psychotherapy and psychological practice, supporting integrative mental health care and guiding the development of personalized therapeutic approaches grounded in embodied experience and contemporary neuroscience. This paper reviews and analyzes state-of-the-art neuroscientific perspectives related to the multi-dimensional foundations and temporo-spatial expansion of the self, in a translational approach linking neuroscience underpinnings of the self to body movement. It considers the self and self-other dynamics and their disturbances as they are woven into personalized and clinically applicable psychotherapeutic understandings and means of cultivating selfhood.

## 2 Action possibilities and generative self-modeling

The PP framework of brain function implies that the way we perceive ourselves and the environment, from the most basic level to the most intricate entanglements of the self, is intertwined with our opportunities for action, or affordances. We sample the world to minimize uncertainty and maintain coherence between internal predictions and sensory inputs. The most important way to reduce prediction error is by acting upon the environment to change the states we want to infer, and the optimal reduction of uncertainty via action is based on the selection of the best option from out of a variety of possible actions (33). Thus, we perceive affordances in our environment, and hence implicitly experience ourselves as agents with the power to perform affordable actions.

Beyond homeostatic adaptation, humans aim to maximize intrinsic or epistemic value, and (enactively) choose the actions that are likely to reduce uncertainty about the states of the body in the changing environment, by curiously seeking surprises and novelty in non-threatening settings (34). The epistemic affordance, or possibilities for action the environment offers, underpins the way we forage for information to achieve self-modeling (35, 36). Affordances (or action possibilities) are formed by the relation between the abilities of an organism and features of the environment, generating a repertoire of potential movements, gestures, and relational actions available to an individual in a given context. Experiences soliciting epistemic affordances determine the direction of future anticipation of affordances (37). The felt states of action readiness manifests as a space of polarities and combinations at the physical, affective and cognitive levels. As the agent prepares to move towards/away, approach/withdraw, and is receptive/defensive to affordances in the environment, these movement tendencies may be consciously felt as pleasant/unpleasant, positive/negative, and relate to affordances the individual likes/dislikes, is attracted/repelled by (38).

Perceived locomotive affordances, reflecting different ways one can move through the environment, form a unique representational space in the brain (39). This strengthens the idea that perception is for action and, inversely, actions create further opportunities for perception. The perception of action-guiding properties of objects in the environment emerges as a novel way of explaining the agentive purposiveness that characterizes how one navigates reality (40). In line with the PP framework, the anticipatory processes involved in action tie action to the particularities of the agent’s affordance space, which is a relational space in that it depends both on the object or environmental feature, and on the agent’s body (41). The recognition of the patient’s bodily features and the identification and incorporation of implicit and explicit expressive movement in psychotherapeutic settings, offer patients opportunities to explore, make meaning of and transform their actions, and thus cultivate their epistemic affordances. Therapeutic change occurs not only through verbal interpretations but through new embodied acts, relational engagements and interpretive action that expand the patient’s generative repertoire of being and acting in the world. Within the psychotherapeutic setting, predictive processes unfold in a dynamic interplay between stability and change, across cycles of alignment (match), surprising expectancy violation (prediction errors\mismatch), and eventual reparation, accompanied by new learning. The therapeutic space may become one where patients can safely generate new actions, postures and relational gestures, counterfactual explorations that produce surprising yet tolerable sensorimotor outcomes. These experiences can update predictive models and epistemic action affordances, enabling the reconstruction of meaning-making processes. The significance of this self-actualization is related to the vital role of the body in selfhood, which is further elaborated on in the next section.

## 3 The bodily foundations of selfhood

The PP account of brain function points to hierarchical models of the self that resonate with its nested-multidimensionality, and the bodily basis for higher forms of self-consciousness (18). The fundamental pre-reflexive layer of the self captures an experiential essence, grounded in bodily signals, which is ecologically and somatically embedded and experienced in the present moment, and viewed in different perspectives as the “proto-self” (42), “minimal self” (43), or “interoceptive self” (44). The fundamental layer of the self evolves through interactions with the environment, by actively extending to the outer body, which is represented as the “extero-proprioceptive self” (44) or the “core self” (42). This dimension relates to the manifestation of the self, which relies on the degree of integration of the self and significant others, and the actual experiences of the self with the external world (45).

Similarly, the space surrounding the body, termed the “kinesphere” (46) or “peripersonal space” (PPS); i.e., the space within reach of the limbs through movement, represents a multisensory and sensorimotor interface mediating body-environment interactions (47). The occupation and use of PPS is a psycho-physical process influenced by internal aspects and environmental factors such as context, gender or culture, and is thus associated with self and relational disturbances and psychopathologies (48, 49). Accordingly, studies have shown that the PPS is influenced by internal and external factors encompassing emotional salience and social cognitive aspects (50), linking peripersonal actions to a vast spectrum of functions including body representation, goal-directed movements, defensive movements, and social interactions (51).

These dynamic bodily features of the self concur with DMT conceptualizations, observations and insights into body-mind mechanisms, which relate to spatial changes in the shape or form of the body and to the quality of movement that embody, enact, influence and express object relations and self-representations. In brief, different DMT paradigms describe the shape of movement that includes changes in the growing or shrinking of body contours, associated with feelings of comfort\discomfort within the relational environment, thus providing the somatic basis for patterns of reaching to or withdrawing from objects, also enabling differentiation between self and objects. These serve as the foundation for the use of range in space, directional movement and three dimensional “shaping” patterns of the body in the PPS (48, 52–54). The qualities of the movement indicate the implicitly enacted rhythmic patterns of change and regulation in muscle tension, i.e. the transitions between free and bound flow that are indicative of the person’s inner needs, affects and feelings; alongside the developing ways of coping with the environment through alterations seen in the use of space, weight and time, i.e. “efforts” (48, 52–54).

In the brain, the ongoing constitution of primary self-dimensions and PPS representations relies mainly on the neural activation and connectivity of brainstem nuclei that include the periaqueductal gray, subcortical regions such as the amygdala, hippocampus, putamen, thalamus, and cerebellum, the somatosensory and sensorimotor cortices, and the salience network anchored in the anterior insula and anterior cingulate cortex (ACC) (55, 56).

While typically characterized as having dynamic and flexible boundaries that are sensitive to motor action factors (57), as well as to affective context (58, 59), PPS disturbances have been found in various disorders of the self (60–62), pointing to its importance in psychotherapeutic evaluations and interventions. Alterations in the shape and qualities of movement within the PPS can be evaluated at the beginning and throughout the therapeutic process. More flexible boundaries, adaptively boosted according to the social situation, can be created by fostering awareness and exploring the perception and use of the PPS, as well as the relationship to the extrapersonal space, i.e., the space far from the body and the PPS of others. This process also involves sensing the dynamic interplay between these external spatial boundaries and the internal milieu, i.e., interoception. Experiencing oneself as both undergoing the sensations and being the source of and initiating actions is fundamental to self-consciousness and underpins senses of ownership and agency (63). Movement-related weak or altered sense of ownership and agency have been associated with disruptions in body image and body schema, in conditions such as depersonalization (64), schizophrenia (65), eating disorders (66), and autism spectrum disorder (67), in which the embodied sense of self becomes fragmented or distorted. The relational context fosters a shared process of predictive multisensory integration linking one’s own actions with the anticipation of the other’s behavior and enabling mutual adjustment during motor interactions (68). Therapists can help patients reorient themselves to the body, a capacity which is for the most part impaired under traumatic circumstances (69), and generally across a range of psychiatric conditions (70). This critical aspects of self-awareness and multisensory integration are further elaborated on in the next section.

## 4 Dwelling in the soma: developing awareness of body and action

The embodied presence within the lived body relies on interoceptive processes; namely, the moment-by-moment mapping of the body’s internal milieu across conscious and unconscious levels, which have been suggested to be the building blocks of the sense of self underlying emotions and feelings (36, 71). Interoception encompasses information about the physiological condition of the entire body, including signals originating from internal organs such as the viscera, heart and lungs, as well as signals from the skin including pleasure from gentle touch, and pain, conveyed by specialized afferent pathways (72, 73).

Within the PP framework, interoception can be seen as the generation of predictive models of the changing sensory conditions within the body that are influenced by sensorimotor and allostatic functions. Signals from the body reach the level of consciousness to regain allostatic balance, thus generating bodily-affective feelings, informing the organism about its bodily needs (e.g., changes in breathing or heart rate due to anxiety, and stress) (74). However, interoception extends beyond immediate physiological responses and adaptive reactions, and has been shown to be deeply interconnected with impulsive processes, affective states and emotional experiences, motivations, and cognition (75). Thus, the impact of interoception is thought to relate to the awareness of oneself as a feeling entity at any given time (76; A. 77).

The neural representation of somatic and visceral information is transmitted through multiple anatomical pathways to several target sites in the brain. These interoceptive pathways include the spinothalamic sympathetic nervous system and vagal parasympathetic nervous system pathways, and brain regions spanning the periaqueductal gray and brainstem centers, subcortical limbic regions, and cortical regions implicated in predicting and representing information from the body, i.e., the somatosensory cortex, the insular cortices, the ACC and ventro-medial prefrontal cortex (78). The posterior insula is thought to be the major site for receiving and processing visceral and other bodily signals and affective states, whereas the anterior insula serves as an association cortex which, as part of the salience network, integrates information from the posterior insula with other continuously perceived sensory input, while detecting salient stimuli to guide motivated behavior through its downstream targets such as the ventral striatum, a central part of the reward network (79). Studies have characterized the anterior insular cortex as a hub mediating interoceptive attention and generating interoceptive predictions (80).

The link between interoception and selfhood has been demonstrated and established in extensive empirical studies (81). The strongest evidence for the role of interoception in emotional and affective experience comes from the striking overlap in neuroanatomical substrates underlying interoception, bodily regulation, and emotional processing (82). Similarly, the PP framework views emotion as “interoceptive inference” (71), and posits that emotional experiences arise from active inference about the presumed causes of interoceptive signals (83). This view holds that interoceptive sense data give rise to affective qualia, which are inferred and categorized within a given context using emotion concepts such as “anger” or “happiness” (82). Alternatively, having a predictive model of the body has been proposed as the basis of the self (77). This self-model depends on the integration of multiple modes of perception, including interoceptive, proprioceptive, somatosensory, and exteroceptive information.

Accordingly, movement and bodily action are integral to emotion construction and regulation (84–86), thus pivotal for psychotherapeutic work. Specific movement qualities are associated with and can induce corresponding emotional states, for example, movements characterized by openness, fluidity, and upward dynamics tend to evoke positive affect, whereas closed, bound, or downward movements are associated with negative emotions (87). Movement therefore becomes both a diagnostic and a transformative tool, enabling access to implicit emotional patterns and offering new embodied routes for emotional expression regulation, and integration.

Therefore, both the awareness of one’s own body and the awareness of one’s own movements are necessary for the experience of selfhood (88). Accordingly, a bidirectional, functional connection between motor actions and interoception was suggested (89). The insula hub was shown to be engaged in both body perception and action awareness, forming a sense of body ownership and of self-agency (56, 90). Embodiment as a basic feature of selfhood is based on the integration of multiple visual, tactile, vestibular, proprioceptive and interoceptive bodily signals (81, 91). Thus, interoception is no longer viewed as an isolated domain, but rather as one that interacts with exteroception, cognition, and action to maintain the organism’s integrity. These interactions generate bodily awareness and self-consciousness, which underlie the formation of coherent bodily self-models (92, 93). Through oscillatory synchrony, predictive coding and multisensory integration in the brain, interoception is anatomically and functionally intertwined with the processing of signals from the external environment (94). The anterior insula subserves key functions of affective processing through monitoring interoceptive signals and connecting them with exteroceptive cues. Such neural processing creates an integrative interface that enables embodiment, emotional mirroring, and higher-order representation (71, 76, 95), which guides attention between internal and external directed cognition (79, 90). Importantly, during the process of integration of multimodal information, the insular cortices are involved in social cognitive processing and self-other differentiation (92).

Atypical interoception, including primary disturbances in implicit interoceptive signaling, as well as aberrant explicit interoceptive and action awareness, have been observed across a range of psychological conditions, suggesting it may be a transdiagnostic factor and common vulnerability in psychopathology (74, 96, 97). Interoceptive dysfunction contributes to disrupted symbolic representations of feeling states, making it difficult to communicate emotions and leading to greater reliance on concrete (i.e., non-symbolic) processes for identifying and regulating them (98).

The association between body awareness and various mental health conditions advocates the importance of integration of its assessments in clinical settings, not only as a diagnostic tool but also as a component of therapeutic strategies (75). Engagement in practices that enhance embodied awareness, such as DMT, could offer a promising avenue for addressing psychopathological conditions and healthcare, by providing valuable insights into the patient’s capabilities to perceive and interpret bodily sensations, and suggesting ways to cultivate them to guide actions (75, 99). The incorporation of action possibilities may facilitate restorative interactions, thus engendering the vital integration and reciprocal interplay between the inner and outer environment. The orientation towards the ongoing salient information originating from the body and expressed in movement, elicits and invites awareness of body sensations and actions that arise, evolve and fluctuate (31). The embodied temporal aspects of these processes are discussed in the following section.

## 5 Embodied subjective temporality in self-modeling

Self-models require a temporal dimension for subjectivity to arise. As conscious beings, people must be able to make inferences about the consequences of their own actions in the future, which relies upon the ability to project oneself through time to select actions that minimize expected or future uncertainty (100). “Temporal thickness” refers to the capacity to make inferences not only about the present moment but also about the past and the future. This ongoing self-evidencing process has a “temporal thickness” involving counterfactual processing; that is, imagining what the next sensory input would be if I were to perform a particular action (101). In this perspective, consciousness is the self-evidencing consequences of *what I could do* (100).

Along convergent lines, Husserl viewed perception as having three temporal aspects, which he coined retention, the immediate present, and protention, which emphasizes the flow through which each moment of protention becomes the retention of the next (102). Subjective time is associated with the conscious self as an enduring entity over time, where processing of time is seen in essence as being embodied. This perceived continuation of time coincides with Winnicott’s moment-by-moment continuous presence that he termed “going on being” (103), as well as conceptualizations such as the “remembered present” and “specious present” (102). Temporality, the subjective experience of time, encompasses more than sensing the time passing; it includes the way experience unfolds as a continuous stream of consciousness rather than as fragmented or discontinuous moments (104). Gallagher has argued that temporality is what explains the directedness of both consciousness and action towards something in the environment (102). Self-movement thus unfolds along a particular trajectory based both on a retention of the body’s configuration in relation to the environment, and an anticipation of where movement is heading next, or the “apprehension of the possibilities or affordances in the present” (p. 13) (102).

Studies in contemporary neuroscience have shown that temporal regularity constitutes a foundational building block for organizing the exteroceptive sensorium, segmenting the world into objects, events, and relationships, and its disruptions may significantly impact perception, emotion and cognition across a range of clinical conditions (105). Moreover, temporal processing is an embodied process that arises from the continuous integration of exteroceptive and interoceptive processes (106), and representations of simultaneity, contingency or duration are rooted in sensorimotor and interoceptive experience. The insular cortex, cerebellum, and supplementary motor area have consistently been shown to be involved in the perceived passage of time, as well as temporal distortions in neurological and psychiatric conditions (107–109). The insular hub integrates exteroceptive and interoceptive signals, to highlight and process emotionally salient moments. It does so by processing temporal cues from changing bodily states and continuously integrating signals across the body, effectively tracking time through internal physiological rhythms. Accordingly, interoceptive processing relies on the influence of temporal oscillations, i.e., cardiac, respiratory and gastric rhythms, on the brain (94). These interoceptive signals shape neural activity and perception (110), prompting and sustaining brain dynamics during an emotional experience (111), which in turn modulates how time is experienced (112). Through its significant roles integrating streams of information from the moving body engaging with the world, the insula shapes our personal, social, and reward-driven actions, our subjective interpretation of our surroundings and our self, which significantly contributes to time perception and our self-consciousness (113).

The central role of subjective temporality underscores the clinical potential of temporal assessments for diagnostic and therapeutic interventions. Therapeutic work should consider the intrinsic subjective temporality of perception and action and be attentive to the patient’s sense of time and duration, the simultaneity or continuity of actions, and the experience of time within the relational context, noticing the pace of experience, cohesion or fragmentation of events and movement synchronization and desynchronization. Therapeutic approaches emphasizing rhythmic movement, temporal attunement, and embodied synchrony may enhance the brain’s capacity for sensory integration and temporal prediction, relational processes that are explored and discussed in greater detail in chapter 8.

The temporally thick generative models suggest that consciousness is fundamentally a memory-dependent process of ‘protension’ or ‘mental time travel’, i.e., the capacity to recall past experiences and simulate possible futures, in embodied agents (16). While “temporal thickness” captures the pre-reflective, embodied organization of moment-to-moment experience, the temporal continuity of self refers to the way we perceive and experience our body and mental states as the same self across time, through mentalization and autobiographical memory. Embodied temporal processes associated with mental time travel abilities, self-referential processes and autobiographical memories, are further addressed in the next section.

## 6 Autobiographical memory and mentalizing entwined with body movement

The sense of self-continuity over time matures into the extended representational dimension of the self, which has been termed the “autobiographical” (42), “narrative” (43) or “mental” self (44) that remembers and reflects on the past, compares it to present experience, envisions and prepares for the future. This dimension relates to reflective self-awareness, metacognitive insight, and more developed social aspects of selfhood. This higher-order self-awareness is necessary for self-identity and autobiographical memory, because it enables the person to differentiate a mental representation of a past event from current experience (114). Intrinsic self-reflection processes, recollection, spontaneous thought, mind-wandering, and mentalizing are mediated by the Default Mode Network (DMN), which sits atop the neural hierarchy, and consists of the medial prefrontal cortex, the posterior cingulate cortex and the adjacent precuneus, bilateral inferior parietal lobes, as well as the medial temporal lobe memory system (including the hippocampus and parahippocampus) (115). The DMN functions as a “sense-making” network that integrates incoming extrinsic (including social) information with prior intrinsic information (memories) to form rich, context-dependent models of situations as they unfold over time (116).

Self-dimensions are connected at their root and dynamically interact, as indicated in studies of brain functionality (117). The ventromedial prefrontal cortex of the DMN was posited to play a major role in integrating physiology, interoception, affect, and self-related processing (118). Volitional action includes temporal dynamics and self-referential processes and was shown to drive the bodily self, sense of ownership and agency through recruitment of the DMN (90). Even the most complex cognitive and emotional mental operations maintain their dependence on the action repertoire of the individual (119) and regions of the DMN appear to be coupled to bodily rhythms (94). Recent evidence suggests, for example, that the act of breathing exerts a substantive, rhythmic influence on perception, emotion, and cognition, through direct modulation of neural oscillations not only in the brainstem but across the cortical hierarchy, including the insular, cingulate and prefrontal cortices (120). The body moves in rhythm with breathing, and breathing rhythmicity is observed in the aforementioned “shape-flow” of the body, i.e., in the growing or shrinking of bodily dimensions in a relational context (52, 120). DMN driven introspective self-reflection, mind-wandering, recall of past events and internal simulation of future events may thus shape and be shaped by the living interactive body and by the interactive encounter with the gestures and actions of other social agents, in a reciprocal manner (116). The anterior insula, mediating interoception, has been proposed to coordinate activity across the DMN and central executive network, by acting as a gatekeeper for executive control, including response inhibition, working memory and cognitive flexibility (121). This view aligns with embodied accounts suggesting that interoceptive predictions shape the dynamic organization of brain activity (122).

Another interesting point in the temporal-spatial context of the self has to do with the role of the hippocampus, an essential hub for episodic memory processing affiliated with the DMN, which is also crucial for spatial cognition. Intriguingly, the same hippocampal circuits that support spatial navigation are also involved in memory, prospection and imagination (123). Considerable research has shown that the hippocampus supports memory formation through its role in representing space (124). The hippocampus was hypothesized to perform a function that relates sequential and relational mechanisms to the actions performed by the body, or to an internalized version of these action sequences (125). Memory can thus be seen as a transmission mechanism gathered from past experiences to guide current and future actions (126). Thus, navigation through either a physical space or an imagined landscape (i.e., mental time travel and planning ahead for an action) maybe accomplished through similar neural mechanisms that may have evolved from the need to predict future needs of the body (119). The ability for mental time travel consists of moving along a cognitive and spatially oriented representation of time, thus allowing for mental travel into the past (memory) and the future (planning) (127). As of early stages of development, reasoning about navigation allows individuals to represent where they are, where they can go and provides an anchor for memories (128). Locations in space overlap social networks, as reflected in the language people use, e.g., feeling “close” to someone or “distant”, standing “above you” or being on “equal footing”, and being in the “same circle” (129). These insights underscore how individual and interpersonal movement can serve as a powerful medium for both the formation and reformation of memory. They further suggest that movement-based therapeutic processes may support the updating, re-contextualization, and integration of past experiences within new spatio-temporal relational contexts.

Unlike autobiographical memories, traumatic memories are not deliberately retrieved as a coherent narrative but instead triggered involuntarily by incoming stimuli that interact with its neural representations, engendering a fragmented re-experience of the event’s sensory and motoric details. Altered activation levels and diminished functional connectivity within and between the DMN and other regions have been identified as a consistent and defining feature of Post-Traumatic Stress Disorder and traumatic re-experiencing (130). Importantly, trauma memories have been suggested to be a transdiagnostic feature of multiple mental health issues (131), highlighting their critical relevance for psychotherapeutic work. Sensorimotor cues can directly reactivate embodied traces of past trauma, because traumatic memories are often stored in bodily sensations and movement patterns. Sensory and motor fragments may require new experiences that contradict past trauma. When old sensations or movements resurface, they can be re-contextualized through present-moment feedback that anchors the body in current time and space, and completing previously inhibited defensive actions can help update the sensorimotor representations of the traumatic event (132). Indeed, essential ingredients of therapeutic change were suggested to involve reactivating old memories, introducing new emotional experiences that can be integrated into those memories during reconsolidation, and then reinforcing the updated memory structure by repeatedly practicing new ways of behaving and experiencing the world across varied contexts (133). Therefore, treatment approaches that utilize emotional expression of differently contextualized sensorimotor reactions, facilitated through the therapist’s attuned presence, coupled with intentional/volitional movement, may encourage the integration of traumatic memories (132, 134). Archaic and traumatic memory traces stored in the sensorimotor system point to their important acknowledgement within the transferential relationship and suggest the potential of using movement improvisation as type of free association (135). The inclusion of bodily movement in therapeutic interventions may act upon and alter ways in which sensory and motor traces are re-experienced in a secure relational setting, which may also foster the contextualization and integration of sensorimotor fragments into meaningful, and possibly verbally accessible autobiographical narratives (136, 137).

Other dysphoric and depressive distortions in memory and affect include excessive mental activity, i.e. rumination, that intensifies the disconnection from embodied experience, creating a recursive loop of alienation from both the body and the external world. This reflects an imbalance in the hierarchical layers of the self, with heightened mental self-referential processing overriding bodily and interoceptive dimensions (138). Incorporating creative movement into psychotherapy may enable the construction and expression of subjective meaningful narratives that cannot be narrated by conventional means (139).

Emotional memory, supported by medial temporal lobe structures involved in emotion and memory, i.e., the amygdala and hippocampus, in conjunction with DMN cortical regions, relies on their coordinated activity during the encoding and retrieval of emotional episodic experiences. Stress-induced emotional arousal, modulated by the neurotransmitter and hormone noradrenaline, results in amplified amygdala-hippocampal circuit, which leads to enhanced encoding and consolidation of the gist of the emotionally arousing information at the expense of contextual details (140). The adaptive prolonged cortisol response, the end product of the hypothalamus-pituitary-adrenal (HPA) axis, modulates hippocampal and prefrontal functions that enable contextualization and coherent memory processing, promoting resilience (141). Sustained post-stress increase in amygdala-hippocampal connectivity was related to attenuated cortisol responsiveness (142), which may underscore stress vulnerability. Movement-related shared experiences have been shown to modulate cortisol responses, having regulatory effects on stress-recovery cycles. Accordingly, infant-mother cortisol synchrony is considered as the physiological manifestation of a dyad’s shared emotional and behavioral experiences (143), facilitating the memory consolidation of these experiences into meaningful emotional knowledge. The embodied neuropsychological mechanisms that lie at the core of these interactive experiences and related therapeutic processes are the topic of the following sections.

## 7 Brains-bodies-minds connection: on kinesthetic empathy and embodied mentalization

The discovery of mirror neurons revealed that other-related multimodal information about sensations, actions, emotions, and communicative messages are mapped onto the beholder’s neural substrates devolved to these first-person self-related processes (144). Mirror neurons, which are activated both while executing and witnessing the actions of others, are widely spread throughout different brain regions, including regions traditionally associated with motor control such as the premotor cortex, supplementary motor cortex, and the cerebellum (i.e., the action observation network). The involvement of mirror mechanisms in the somatosensory cortex prompted the realization that the brain of the witness not only enables the performance of similar actions but also the somatosensory representations of what it would feel like to move in the observed way (145). These processes entail neural representations of motor actions and somatosensory feedback. Accumulating neuroimaging studies have shown that the salience network, i.e., the anterior insula and ACC, critical for interoceptive awareness, becomes reactivated by the same emotional state witnessed in others, a mechanism that allows for an understanding of others’ actions as emotional events, through simulated shared body states, i.e., *embodied simulation* (144, 146). This implies that people use their own bodily experiences and their processing and representations to understand both their own and others’ intentions and emotional experiences (147). This is in line with the notion that body and action awareness contribute to subjective emotional feeling states that can also guide empathic understanding of the emotions of others, thus serving as a crossmodal target for interventions to enhance interpersonal skills.

Importantly, it is the quality of movement that is critical for linking action and emotion. Closely linked to DMT conceptualizations, “vitality forms”, the how-dimension of the unfolding of actions, detected based on movement dynamics, related to the temporal profile, force, space and direction, matching shape and effort and providing an access to unconscious past relational experience (148), were shown to selectively activate the central insula and middle cingulate cortex when an action is performed, imagined, and observed in others (149). The activity of the insula while processing affective information was recently shown to precede and foreshadow action, modulating the premotor cortex (150). Findings also indicate that vitality forms that are characteristic of everyday interactions are atypical in neurodivergent individuals, such as in autism, both in their expression and recognition, thus preventing fluid, preverbal social alignment (151–153). Psychotherapeutic relational mirroring and rhythmic movement interventions, such as the ones used in DMT, were suggested to initiate and clarify these forms and support their predictive processing and then build toward sharing more nuanced actions (154).

The use of the *embodied simulation* mechanism enables *kinesthetic empathy* to arise, i.e., the ability to feel and understand the experience of others with and through movement (155). This is central to DMT and is considered one of its major contributions to psychotherapy. It refers to the therapists’ awareness of their corporal feelings and sensation of movement that arise in response to their patients’ body movements or postures, which enable therapists to infer and respond to their patient’s emotional state (156). Kinesthetic empathy may be fostered through affective attunement, somatic mirroring and synchronous movement. The hormone and neurotransmitter oxytocin has been suggested to underlie this kinesthetic dimension of empathy fostering interpersonal synchrony (157). Social stressors, such as social isolation, exposure to social trauma and loss, are often paralleled by maladaptation of the oxytocin system, whereas restoring this system functioning can reinstate socio-emotional allostasis (158).

It is important to note that although influential, the mirror-neuron framework has been challenged for offering an overly narrow and often unsupported account of how humans understand others’ actions. Recent critiques point out that mirror neuron brain areas contribute to low-level processing of observed actions (e.g., distinguishing types of grip), and copying of body movement topography, but not to high-level action interpretation (e.g., inferring actors’ intentions), and argue that social and action understanding relies more on mechanisms such as associative learning (159).

In addition to *embodied simulation* mirror mechanisms, i.e., shared representations of firsthand and vicarious experiences of affective states, empathy entails reflective participation enabling mental state attribution, which relies on symbolic and abstract processing. Affective and cognitive understanding of the other involves the activation of a theory of mind or “mentalizing” network that primarily taps the temporo-parietal junction (TPJ) and regions of the DMN (160). This mentalizing process is bodily-anchored. The functioning of the DMN is deeply rooted in interoceptive and sensorimotor processes (94, 116). The TPJ has been shown to be implicated in bodily self-location, ownership and self-identification (161), in visuo-spatial perspective taking, sense of agency, mental imagery of one’s own body, and biological motion, as well as in distinguishing between self and other, both mentally and behaviorally in imitation of action experiments (44, 162, 163). Multisensory disintegration at the TPJ may result in out-of-body experiences (164). By navigating between representations of mental states of self and others, the mentalizing network is involved in the attribution of beliefs, emotions and intentions (165, 166), using episodic prospection, facilitating predictive processing (33). By integrating inputs from multiple brain regions through its functional connections with the sensory cortices, hippocampus, and salience network, the DMN is thought to generate both visceral and motor embodied simulations of others’ actions and mental states (167).

The embodied mentalization of the psychotherapist can drive processes of working through unconscious, unformulated or unrepresented states, in which implicit automatic bodily responses that have yet to be mentally represented can be recognized, using embodied narration, symbolic formation and metaphoric work. Such processes may facilitate the functional crosstalk and integration of brain networks, which can prompt experience-dependent neuroplasticity (168).

Embodied attunement within the psychotherapeutic relationship forms the basis for emotional communication and regulation of the bond between the patient and the empathic therapist (169–171). Embodied “dialectical attunement” grounds higher-order abstract thinking, which is considered a re-enactment of the internalized primary dialogue through which individuals acquired the concepts in the first place (172, 173). The therapist helps generate top-down predictive inferences on the causes of bodily signals by resonating with the patient’s experience and by gradually training the patient to mentalize via embodied reflective discourse.

The “relational dance” between patient and therapist (174) promotes the development of the patient’s “inner witness” (175), through the capability to notice, observe and mentalize bodily sensations, associations, images, feelings or thoughts which arise moment-to-moment (176). This coincides with the integration and crosstalk between neural networks associated with the different self-dimensions, e.g., subcortical regions such as the brainstem, amygdala and cerebellum, somatosensory and sensorimotor cortices, salience network, and DMN. In order to do so, the mover needs the therapist as a holding, seeing, and mentalizing “outer witness” that can use somatic countertransference and the function of self-observation, being an “inner witness” to her/himself, fluctuating attention between the internal experience and the awareness of the moving other, further facilitating a reflective dialogue. This is why developing an “inner witness” is needed before becoming an “outer witness” to others (174), both of which functions are cultivated in DMT training and practice (177).

Through empathic therapeutic relationships, predictive models about the availability and support of another with whom one can share meaning are created. Such interpersonal processes relate to the mechanisms of synchrony.

## 8 Brain-to-brain coupling linked with movement synchronicity

When experiencing meaningful moments of togetherness and affect sharing, interpersonal neuro-physio-psychological synchrony can emerge (178). Second-person neuroscience has operationalized and empirically demonstrated synchronization patterns between people at the neural, physiological, and behavioral level via multi-brain recording approaches using hyperscanning techniques that simultaneously record activity from two or more interacting individuals. Coordinated or synchronized brain activation between two people or more, i.e., Interpersonal Neural Synchrony (INS), has been associated with the mirror mechanism, where synchronizing the actions of one individual with the perceptions of another and vice- versa may then lead to interpersonal synchronization of brain oscillations (178).

Social and emotional alignment was shown to be interrelated to the synchronization of movement, as processes that rely on shared neural networks (179). This spontaneous and dynamic synchronization between individuals was found to be present in large-scale brain networks related to sensory perception, attentional salience and embodied simulation, reward processing, and mentalizing processes (180). Robust brain regional correlates of biobehavioral synchrony were found in the TPJ, involved in bodily-anchored self-other processing, and the ventromedial prefrontal cortex, a hub of integration promoting self and social processing (180). INS may be directly mediated by the person-to-person communicative signals through which the interaction is conveyed (e.g., eye contact, facial expression, bodily gestures and movements, and vocal prosody) (181).

Serving as an anchor to human interaction, INS has been proposed as a neurobiological mechanism in psychotherapy that makes it possible to infer the patient’s inner states and facilitate mutual understanding and emotional exchange (182), as an experience-dependent phenomenon (183). Similarly, nonverbal movement synchrony has recently been identified as a central aspect of psychotherapy facilitating the therapeutic alliance (19, 22, 23). Through mutual attention, and behavioral mirroring, INS has been shown to be indicative of empathic relationships, bonding and rapport (184) that is also associated with the development of resilience (185).

Shared interactive moments within psychotherapeutic relationships can induce INS, when fostered by rhythmic movement synchronization, a basic element of DMT (25), as therapists attune to and share the physical and emotional form and quality of their patients’ movement. Synchronization between patient and therapist was shown to be accompanied by physiological synchrony of autonomic signals, as observed during psychotherapy sessions at the hormonal level, i.e. oxytocin (186), heart rate and respiration (187), and skin conductance (188). Oxytocin has been linked to both enhanced INS and behavioral synchrony (189), and its release during interpersonal interaction may underlie the affiliative consequences of synchronous movement. A recent analysis has strengthened the central role intersubjective synchrony in psychotherapy, fostering a stronger alliance and deeper connectedness, thereby promoting therapeutic progress (190). The study highlighted the layered dynamics of synchrony, showing that physiological synchrony facilitates moment-to-moment affective attunement, whereas behavioral synchrony supports sustained relational engagement over time. Therapist-led nonverbal synchrony has been specifically linked to a stronger therapeutic alliance and improved treatment outcomes.

Interpersonal communication depends on shared predictive models (191, 192). Indeed, INS was suggested to entrain shared PP, where the oscillatory synchrony between people may bind potentially disruptive “free energy” together (193). This implies that when people engage in meaningful interpersonal engagement by interacting on a physical, emotional and mental level, they not only predict each other, but (en)actively entrain their perceptual and motor states, thus forming a shared entity of active inference in embodied creative ways (18). Inferential models may become similar over time, thus enabling mutual understanding to develop, a process that was linked with interbrain plasticity, or the change in interbrain coupling over time in neural networks associated with social learning, e.g., via the mentalizing network, hippocampus, action observation network, and salience network (194). Through its functional connections, the DMN representation of the other’s behavior and underlying mental states may result in a synchronized response facilitating shared understanding and empathy (167). Such moments in which the minds of both parties intersect and co-construct a shared experience that can be perceived by each participant, have been suggested to relate to the sharing of “vitality forms” of movement (195).

By contrast, mental disorders have been associated with a lesser ability to achieve synchrony at the neural, behavioral and psychological levels (196, 197). Restoring interpersonal synchrony emerges as a key therapeutic aim, whereby therapists and patients may become dynamically coupled across sensory-motor, affective, and neural domains (198). Indeed, recurring opportunities for synchronization in therapy may lead to plastic and lasting changes in a person’s overall ability to synchronize (168, 199). In psychotherapy, and particularly in movement-based approaches, such synchrony can be used to explore and reshaped dysfunctional interaction patterns. Attuned therapists and patients spontaneously synchronize in their movement behavior, and aspects of their voice, along with neurophysiological processes (200), promoting emotional processing and regulation (182). Throughout a therapeutic interaction therapists move in and out of sync with the patients, from states of match to mismatch and reparation, enabling the therapeutic relationship to be curative, so that a lower trait ability to sync with others can be subject to state-like manipulation (201). Therapists can notice and intentionally work with movement synchrony, breath, rhythm, pacing, distance or proximity and variations in peripersonal and extrapersonal space, direction, the use of power, and interoceptive awareness scaffolding to connect with their patients, reduce uncertainty and induce new learning, experiencing moments of co-regulation. These moments of synchrony are fundamental to the dialectical process between self and other that shapes the sense of self (45). Through these embodied dynamics patients can sense, enact, and reflect on their habitual relational strategies, gaining insight into maladaptive patterns while experiencing new possibilities for connection, agency, and mutual regulation.

## 9 Summary and conclusions

Current views of brain function anchor the link and bridge the divide between the somatic and the psychic, by revealing the multifaceted clinical aspects of the living moving body, as applied in DMT and applicable to psychotherapy in general. The primacy of body movement in the constitution and development of the self, and at every level of mental functioning and dysfunctioning, makes it a vital source of information and intervention.

Recognition of patients’ movement repertoire and use of the PPS, as well as its broadening, can be a critical part of the assessment and treatment processes. Embodied mentalization, observation and analysis of the shape and qualities of movement, while linking them to internal sensations, emotions and mental processes of both patient and therapist, may help therapists better understand their patients’ functioning and inform the transference-countertransference matrix. The epistemic affordance of the therapeutic encounter can be manifested through its actualization by offering a wide range of action possibilities that enable the generation of updated models of the self within the relational context.

Psychotherapy that cultivates awareness of the body and action prompts individuals’ ability to perceive, integrate and interpret their bodily cues as feelings and cognitions, and consequently better regulate their emotions. Interpersonal communicative methods based on kinesthetic empathy and embodied resonance using bodily means of attunement, mirroring, and synchronous movement, meaningfully coincide with the relational neural mirroring mechanisms enabling embodied simulation, and neurophysiological synchronization. The therapy process, encompassing both experiential and reflective meaning-making processes, can encourage embodied mentalization and integration of the self-dimensions.

Psychotherapeutic interventions can build up self-awareness involving inferential processes with counterfactual depth. The experiential body can thus be entwined with the “temporal thickness” of the specious present, thus raising awareness to the subjective experience of time and further developing the capacity for mental time travel and the consolidation of long-term autobiographical episodic memories. Embodied attention and the accumulating experiences of movement possibilities can ground inferences about the (relational) consequences of action. Autobiographical narratives that might never have been narrated by conventional means can be reassembled, given meaning and sequential temporal and spatial properties, reconsolidating trauma memories, and at the same time creating shared emotionally corrective narratives. The psychotherapeutic use of body movement involves vital transitions between immersion (first-person perspective) and distancing (third-person perspective), allowing for a more flexible, adaptive, integrated self experience.

Psychotherapy mobilizes active inference in the context of intimate relationships by enabling a trustful atmosphere and by “loaning” the therapist’s brain functions, creating a “reverie” that can provide a way to enter the predictive moment (2). Moments of creative uncertainty may emerge and through them the need for active exploration, innovation and generative possibilities. Psychotherapy can encompass two strategies to engage predictive processing neurodynamics. The first relates to the “bottom-up” promotion of prediction errors, by opening up opportunities for action within corrective relational emotional experiences, increasing “free energy”, and sampling new sensorimotor input through action (active/enactive inference). The second regards the “top-down” shaping of the internal models of the body in the world (prediction signals) through reflective meaning-making processes. These are interlaced in the therapeutic process within which subjects become meaning-makers who dynamically co-evolve with the world they inhabit and predict (139).

The broad body-oriented range of intervention techniques drawing on implicit relational knowing and embodied participatory sense making, enables practitioners to work at all levels of mental functioning. Primary self-disturbances may require intensified vitalizing interventions to enliven self-awareness and the perception of body boundaries and PPS, thus engaging patients in interpersonal exchange (202, 203). Higher level self-related processes require symbolic transformation, allowing psychic reality to transform “primary psychic matter” (‘free energy’) into a matter endowed with reflexivity and subjectivity (18). The use of imagery, symbolism, metaphoric language and reflective dialogue facilitates meaning-making and body-mind connections, thus nurturing the formation of increasingly complex, embodied and coherent internal (generative) representations, models of the embodied, embedded, enactive, and extended self in the world.

Future directions and strategies to further strengthen evidence-based neuroscience research on the psychotherapeutic use of body-oriented interventions in conjunction with brain circuitry and brain-body-mind interactions are highly valuable. Future studies may seek to advance analytical approaches to uncover neural synchronization mechanisms related to the similarity of shared behavioral creative experiences (204). The use of mobile technology such as Mobile Brain-Body Imaging (MoBI) in a hyperscanning setting is at the forefront of advancing our understanding of the intricate interaction between the brain, the body, and the social environment (139, 205). MoBI has been used to investigate the multi-modal nature of expressive movement, such as dance, using real-time changes in brain dynamics and behavior, thus paving the way to explore the neural computations underlying complex motor action, timing and memory (206). In order to assess psychotherapeutic outcomes, the combination of neural biomarkers from MoBI recordings and patients’ subjective reported experiences in a mixed-method research design could provide a more holistic integrative approach for examining the interplay between neuronal dynamics and phenomenological accounts of self-experience (138, 207, 208). Moreover, hyperscanning research that tracks INS over time, integrated with behavioral and psychological observations, can help meet the need for longitudinal designs and highlight their potential in studying mechanisms underlying therapeutic change (168, 209, 210).

Grounded in neuroscience perspectives on the self and self-other relations, this article aimed to shed light on the ways in which body- and movement-informed psychotherapy can act upon, manifest, and cultivate the multidimensional aspects of selfhood. By recognizing the multifaceted meanings of movement both as a vital source of information and a catalyst for change, psychotherapy can foster body-mind integration through creatively and relationally minding the moving self.

## References

[1] Overgaard M Preston C Aspell J . The self, its body and its brain. Sci Rep. (2023) 13:12761. doi: 10.1038/s41598-023-39959-w, PMID: 37550400 PMC10406811

[2] Vaisvaser S . The embodied-enactive-interactive brain: bridging neuroscience and creative arts therapies. Front Psychol. (2021) 12:634079. doi: 10.3389/fpsyg.2021.634079, PMID: 33995190 PMC8121022

[3] Nord C Garfinkel S . Interoceptive pathways to understand and treat mental health conditions. Trends Cogn Sciences. (2022) 6:499–513. doi: 10.1016/j.tics.2022.03.004, PMID: 35466044

[4] Fuchs T . The circularity of the embodied mind. Front Psychol. (2020) 11:1707. doi: 10.3389/FPSYG.2020.01707, PMID: 32903365 PMC7434866

[5] Fabiani M . The embodied brain. Psychophysiology. (2015) 52:1–5. doi: 10.1111/psyp.12381, PMID: 25537620

[6] Gallagher S Hutto D Slaby J Hospital P . The brain as part of an enactive system. Behav Brain Sci. (2013) 36:421–2. doi: 10.1017/S0140525X12002105, PMID: 23883750

[7] Parada FJ Grasso-Cladera A Rossi A Soto-Icaza P Arenas-Pérez M Errázuriz MC . Applied human neuroscience: Fostering and designing inclusive environments with the 3E-Cognition perspective. Eur J Neurosci. (2024) 60:4148–68. doi: 10.1111/EJN.16463, PMID: 39001625

[8] Friston K . The free-energy principle: A unified brain theory? Nat Rev Neurosci. (2010) 11:127–38. doi: 10.1038/nrn2787, PMID: 20068583

[9] Hutchinson JB Barrett LF . The power of predictions: an emerging paradigm for psychological research. Curr Dir psychol Sci. (2019) 28:280–91. doi: 10.1177/0963721419831992, PMID: 31749520 PMC6867616

[10] Bastos A Usrey W Adams R Mangun G Fries P Friston K . Canonical microcircuits for predictive coding. Neuron. (2012) 4:695–711. doi: 10.1016/j.neuron.2012.10.038, PMID: 23177956 PMC3777738

[11] Parr T Friston KJ . Generalised free energy and active inference. Biol Cybernetics. (2019) 113:495–513. doi: 10.1007/S00422-019-00805-W, PMID: 31562544 PMC6848054

[12] Pezzulo G Parr T Friston K . The evolution of brain architectures for predictive coding and active inference. Philos Trans R Soc B. (2022) 377:20200531. doi: 10.1098/RSTB.2020.0531, PMID: 34957844 PMC8710884

[13] Cieri F Esposito R . Psychoanalysis and neuroscience: the bridge between mind and brain. Front Psychol. (2019) 10:1983/FULL. doi: 10.3389/FPSYG.2019.01983/FULL, PMID: 31555159 PMC6724748

[14] Kirchhoff M Parr T Palacios E Friston K Kiverstein J . The Markov blankets of life: autonomy, active inference and the free energy principle. J R Soc Interface. (2018) 15:20170792. doi: 10.1098/RSIF.2017.0792, PMID: 29343629 PMC5805980

[15] Hohwy J . The self-evidencing brain. Nous. (2016) 50:259–85. doi: 10.1111/NOUS.12062

[16] Limanowski J Friston K . Seeing the Dark”: Grounding phenomenal transparency and opacity in precision estimation for active inference. Front Psychol. (2018) 9:643. doi: 10.3389/fpsyg.2018.00643, PMID: 29780343 PMC5945877

[17] Solms M Friston K . How and why consciousness arises: Some considerations from physics and physiology. J Consciousness Stud. (2018) 25:202–38.

[18] Vaisvaser S . Meeting the multidimensional self: fostering selfhood at the interface of Creative Arts Therapies and neuroscience. Front Psychol. (2024) 15:1417035. doi: 10.3389/fpsyg.2024.1417035, PMID: 39386142 PMC11461312

[19] Gregorini C De Carli P Parolin LAL Tschacher W Preti E . Potential role of nonverbal synchrony in psychotherapy: A meta-analysis. Counselling Psychother Res. (2025) 25:e12885. doi: 10.1002/CAPR.12885

[20] Tschacher W Giersch A Friston K . Embodiment and schizophrenia: A review of implications and applications. Schizophr Bull. (2017) 43:745–53. doi: 10.1093/SCHBUL/SBW220, PMID: 28338892 PMC5472128

[21] Costa-Cordella S Grasso-Cladera A Parada FJ . The future of psychotherapy research and neuroscience: Introducing the 4E/MoBI approach to the study of patient–therapist interaction. Rev Gen Psychol. (2024) 28:143–65. doi: 10.1177/10892680231224399

[22] Angeletti LL Scalabrini A Galassi F Northoff G . The Relational self in psychotherapy: intersubjective and intrasubjective synchrony in interpersonal space. In: Shapiro Y , editor. Psychodynamic psychotherapy: A global perspective (2024) New York: Nova Science.

[23] Høgenhaug SS Kongerslev MT Kjaersdam Telléus G . The role of interpersonal coordination dynamics in alliance rupture and repair processes in psychotherapy—A systematic review. Front Psychol. (2023) 14:1291155/XML/NLM. doi: 10.3389/FPSYG.2023.1291155/XML/NLM, PMID: 38239459 PMC10794593

[24] Levy F . Dance/movement therapy: A healing art. In: American Journal of Dance Therapy, 2nd Edition. National Dance Association of AAHPERD, Reston, VA (2005). doi: 10.1007/S10465-008-9048-9

[25] Chaiklin S Schmais C . The Chace approach to dance therapy. In: Sandel S Chaiklin S Lohn A , editors. Foundations of dance/movement therapy: The life and work of Marian Chace. Columbia: Marian Chace Memorial Fund of the American Dance Therapy Association (1993). p. 75–9.

[26] Samaritter R . Dance movement therapy. In: The Routledge International Handbook of Embodied Perspectives in Psychotherapy: Approaches from Dance Movement and Body Psychotherapies. New York: Routledge (2019).

[27] Fancourt D Finn S . What is the evidence on the role of the arts in improving health and well-being? A scoping review (2019). Regional Office for Europe: World Health Organization. Available online at: https://apps.who.int/iris/handle/10665/329834 (Accessed September 27, 2025). 32091683

[28] Aithal S Moula Z Karkou V Karaminis T Powell J Makris S . A systematic review of the contribution of dance movement psychotherapy towards the well-being of children with autism spectrum disorders. Front Psychol. (2021) 12:719673. doi: 10.3389/FPSYG.2021.719673, PMID: 34744883 PMC8564751

[29] Karkou V Aithal S Zubala A Meekums B . Effectiveness of dance movement therapy in the treatment of adults with depression: A systematic review with meta-analyses. Front Psychol. (2019) 10:936. doi: 10.3389/FPSYG.2019.00936, PMID: 31130889 PMC6509172

[30] Koch SC Riege RFF Tisborn K Biondo J Martin L Beelmann A . Effects of dance movement therapy and dance on health-related psychological outcomes. A meta-analysis update. Front Psychol. (2019) 10:1806. doi: 10.3389/FPSYG.2019.01806, PMID: 31481910 PMC6710484

[31] Millman LSM Terhune DB Hunter ECM Orgs G . Towards a neurocognitive approach to dance movement therapy for mental health: A systematic review. Clin Psychol Psychother. (2021) 28:24–38. doi: 10.1002/CPP.2490, PMID: 32539160

[32] Wu CC Xiong HY Zheng JJ Wang XQ . Dance movement therapy for neurodegenerative diseases: A systematic review. Front Aging Neurosci. (2022) 14:975711. doi: 10.3389/FNAGI.2022.975711, PMID: 36004000 PMC9394857

[33] Lehmann K Bolis D Friston K Schilbach L Ramstead MJD Kanske P . An active-inference approach to second-person neuroscience. Perspectives psychol Sci. (2023) 19(6):931–51. doi: 10.1177/17456916231188000, PMID: 37565656 PMC11539477

[34] Clark A . A nice surprise? Predictive processing and the active pursuit of novelty. Phenomenology Cogn Sci. (2018) 17:521–34. doi: 10.1007/S11097-017-9525-Z

[35] Köster M Kayhan E Langeloh M Hoehl S . Making sense of the world: infant learning from a predictive processing perspective. Perspect psychol Sci. (2020) 15:562–71. doi: 10.1177/1745691619895071, PMID: 32167407 PMC7243078

[36] Picard F Friston K . Predictions, perception, and a sense of self. Neurology. (2014) 83:1112–8. doi: 10.1212/WNL.0000000000000798, PMID: 25128179 PMC4166359

[37] Van Dijk L Rietveld E . Situated anticipation. Synthese. (2021) 198:349–71. doi: 10.1007/S11229-018-02013-8, PMID: 33583961 PMC7822785

[38] Kiverstein J Miller M Rietveld E . The feeling of grip: novelty, error dynamics, and the predictive brain. Synthese. (2019) 196:2847–69. doi: 10.1007/S11229-017-1583-9

[39] Bartnik CG Sartzetaki C Sanchez AP Molenkamp E Bommer S Vukšić N . Representation of locomotive action affordances in human behavior, brains, and deep neural networks. Proc Natl Acad Sci. (2025) 122:e2414005122. doi: 10.1073/PNAS.2414005122, PMID: 40504155 PMC12184334

[40] Heft H Sinha C Kiverstein J Heras-Escribano M De Pinedo-García M . Affordances and landscapes: Overcoming the nature–culture dichotomy through niche construction theory. Frontiers Psychol. (2018) 8:2294. doi: 10.3389/FPSYG.2017.02294/FULL, PMID: 29375426 PMC5767241

[41] Gallagher S Aguda B . Anchoring know-how: Action, affordance, and anticipation. J Consciousness Stud. (2020) 27:11–37.

[42] Damasio A . Self comes to Mind: Constructing the Conscious Brain. London: William Heinemann (2010).

[43] Gallagher S . Philosophical conceptions of the self: Implications for cognitive science. Trends Cogn Sci. (2000) 4:14–21. doi: 10.1016/S1364-6613(99)01417-5, PMID: 10637618

[44] Qin P Wang M G Northoff G . Linking bodily, environmental and mental states in the self—A three-level model based on a meta-analysis. Neurosci Biobehav Rev. (2020) 115:77–95. doi: 10.1016/j.neubiorev.2020.05.004, PMID: 32492474

[45] Scalabrini A Mucci C Northoff G . The nested hierarchy of self and its trauma: In search for a synchronic dynamic and topographical re-organization. Front Hum Neurosci. (2022) 16:980353. doi: 10.3389/fnhum.2022.980353, PMID: 36118976 PMC9478193

[46] Laban R . The language of movement: a guidebook to Choreutics. Ullmann L , editor. London: MacDonald and Evans (1966).

[47] Serino A . Peripersonal space (PPS) as a multisensory interface between the individual and the environment, defining the space of the self. Neurosci Biobehav Rev. (2019) 99:138–59. doi: 10.1016/j.neubiorev.2019.01.016, PMID: 30685486

[48] Bartenieff I Lewis D . Body movement: Coping with the environment. In: Body movement: coping with the environment. Routledge (1980).

[49] Ylla Boix R Panhofer H . The kinesphere: a systematised literature review. In: Body, movement and dance in psychotherapy (2024) 20(1), 4–21. doi: 10.1080/17432979.2024.2355133

[50] Basile GA Tatti E Bertino S Milardi D Genovese G Bruno A . Neuroanatomical correlates of peripersonal space: bridging the gap between perception, action, emotion and social cognition. Brain Structure Funct. (2024) 229:1047–72. doi: 10.1007/S00429-024-02781-9, PMID: 38683211 PMC11147881

[51] Geers L Coello Y . The relationship between action, social and multisensory spaces. Sci Reports. (2023) 13:202. doi: 10.1038/s41598-023-27514-6, PMID: 36604525 PMC9814785

[52] Amighi JK Loman S Sossin KM . The meaning of movement: embodied developmental, clinical, and cultural perspectives of the kestenberg movement profile. In: The Meaning of Movement: Embodied Developmental, Clinical, and Cultural Perspectives of the Kestenberg Movement Profile (2018). p. 1–362. doi: 10.4324/9781351038706. New York: Routledge.

[53] Koch SC Sossin KM . Kestenberg movement analysis. In: Body–Language–Communication: An International Handbook on Multimodality in Human Interaction. DeGruyter, Berlin (2013).

[54] Loman S Merman H . The KMP: A tool for dance/movement therapy. Am J Dance Ther. (1996) 18:29–52. doi: 10.1007/BF02360220

[55] Scalabrini A Cavicchioli M Benedetti F Mucci C Northoff G . The nested hierarchical model of self and its non-relational vs relational posttraumatic manifestation: an fMRI meta-analysis of emotional processing. Mol Psychiatry. (2024) 29(9):2859–72. doi: 10.1038/s41380-024-02520-w, PMID: 38514803

[56] Seghezzi S Giannini G Zapparoli L . Neurofunctional correlates of body-ownership and sense of agency: A meta-analytical account of self-consciousness. Cortex. (2019) 121:169–78. doi: 10.1016/j.cortex.2019.08.018, PMID: 31629195

[57] Làdavas E Serino A . Action-dependent plasticity in peripersonal space representations. Cogn Neuropsychol. (2008) 25:1099–113. doi: 10.1080/02643290802359113, PMID: 18726788

[58] de Haan AM Smit M Van Der Stigchel S Dijkerman HC . Approaching threat modulates visuotactile interactions in peripersonal space. Exp Brain Res. (2016) 234:1875–84. doi: 10.1007/S00221-016-4571-2, PMID: 26894891 PMC4893051

[59] Ferri F Tajadura-Jiménez A Väljamäe A Vastano R . Emotion-inducing approaching sounds shape the boundaries of multisensory peripersonal space. Neuropsychologia. (2015) 70:468–75. doi: 10.1016/j.neuropsychologia.2015.03.001, PMID: 25744869

[60] Asada K Akechi H Kikuchi Y Tojo Y Hakarino K Saito A . Longitudinal study of personal space in autism. Child Neuropsychol. (2025) 31:119–27. doi: 10.1080/09297049.2024.2337753, PMID: 38578305

[61] Noel J Cascio C Wallace M Park S . The spatial self in schizophrenia and autism spectrum disorder. Schizophr Research. (2017) 179:8–12. doi: 10.1016/j.schres.2016.09.021, PMID: 27650196 PMC5219859

[62] Rabellino D Frewen PA McKinnon MC Lanius RA . Peripersonal space and bodily self-consciousness: implications for psychological trauma-related disorders. Front Neurosci. (2020) 14:586605. doi: 10.3389/fnins.2020.586605, PMID: 33362457 PMC7758430

[63] Gallese V Sinigaglia C . The bodily self as power for action. Neuropsychologia. (2010) 3:746–55. doi: 10.1016/j.neuropsychologia.2009.09.038, PMID: 19835895

[64] Alekseeva V Ciaunica A . It is movement all the way down: broken rhythms and embodied selfhood in depersonalization. Behav Sci. (2025) 15:1090. doi: 10.3390/bs15081090, PMID: 40867447 PMC12383108

[65] Klaver M Dijkerman HC . Bodily experience in schizophrenia: factors underlying a disturbed sense of body ownership. Front Hum Neurosci. (2016) 10:305. doi: 10.3389/fnhum.2016.00305, PMID: 27378895 PMC4911393

[66] Carey M Preston C . Investigating the components of body image disturbance within eating disorders. Front Psychiatry. (2019) 10:635. doi: 10.3389/fpsyt.2019.00635, PMID: 31620027 PMC6759942

[67] Kaganoi S Sawada K Sota S Nagano S Takahashi H Kazui H . Impairments in the sense of self in children with autism spectrum disorder. Psychiatry Res. (2025) 352:116668. doi: 10.1016/j.psychres.2025.116668, PMID: 40795455

[68] Fanghella M Era V Candidi M Palermo L Boccia M . Interpersonal motor interactions shape multisensory representations of the peripersonal space. Brain Sci. (2021) 11:255. doi: 10.3390/brainsci, PMID: 33669561 PMC7922994

[69] Tsur N Defrin R Lahav Y Solomon Z . The traumatized body: Long-term PTSD and its implications for the orientation towards bodily signals. Psychiatry Res. (2018) 261:281–9. doi: 10.1016/j.psychres.2017.12.083, PMID: 29329049

[70] Brewer R Murphy J Bird G . Atypical interoception as a common risk factor for psychopathology: A review. Neurosci Biobehav Reviews. (2021) 130:470–508. doi: 10.1016/j.neubiorev.2021.07.036, PMID: 34358578 PMC8522807

[71] Seth AK . Interoceptive inference, emotion, and the embodied self. Trends Cogn Sci. (2013) 17:565–73. doi: 10.1016/j.tics.2013.09.007, PMID: 24126130

[72] Ceunen E Vlaeyen JWS Van Diest I . On the origin of interoception. Front Psychol. (2016) 7:743. doi: 10.3389/fpsyg.2016.00743, PMID: 27242642 PMC4876111

[73] Craig AD . How do you feel? Interoception: The sense of the physiological condition of the body. Nat Rev Neurosci. (2002) 13(4):500–5. doi: 10.1038/nrn894, PMID: 12154366

[74] Berntson G Khalsa S . Neural circuits of interoception. Trends Neurosciences. (2021) 44:17–28. doi: 10.1016/j.tins.2020.09.011, PMID: 33378653 PMC8054704

[75] Solano Durán P Morales JP Huepe D . Interoceptive awareness in a clinical setting: the need to bring interoceptive perspectives into clinical evaluation. Front Psychol. (2024) 15:1244701. doi: 10.3389/fpsyg.2024.1244701, PMID: 38933585 PMC11199726

[76] Craig AD . How do you feel - now? The anterior insula and human awareness. Nat Rev Neurosci. (2009) 10:9–70. doi: 10.1038/nrn2555, PMID: 19096369

[77] Seth A Tsakiris M . Being a beast machine: The somatic basis of selfhood. Trends Cogn Sciences. (2018) 22:969–81. doi: 10.1016/j.tics.2018.08.008, PMID: 30224233

[78] Feldman MJ Bliss-Moreau E Lindquist KA . The neurobiology of interoception and affect. Trends Cogn Sci. (2024) 28:643–61. doi: 10.1016/J.TICS.2024.01.009, PMID: 38395706 PMC11222051

[79] Uddin L . Salience processing and insular cortical function and dysfunction. Nature Rev Neuroscience. (2015) 16:55–61. doi: 10.1038/nrn3857, PMID: 25406711

[80] Wang X He Y Lu K Deng C Qiao X Hao N . How does the embodied metaphor affect creative thinking? NeuroImage. (2019) 202:116114. doi: 10.1016/j.neuroimage.2019.116114, PMID: 31442486

[81] Park HD Blanke O . Coupling inner and outer body for self-consciousness. Trends Cogn Sciences. (2019) 23:377–88. doi: 10.1016/j.tics.2019.02.002, PMID: 30826212

[82] Quigley K Kanoski S Grill W Barrett L Tsakiris M . Functions of interoception: From energy regulation to experience of the self. Trends Neurosci. (2021) 44:29–38. doi: 10.1016/j.tins.2020.09.008, PMID: 33378654 PMC7780233

[83] Seth AK Friston KJ . Active interoceptive inference and the emotional brain. Philos Trans R Soc B: Biol Sci. (2016) 371:20160007. doi: 10.1098/RSTB.2016.0007, PMID: 28080966 PMC5062097

[84] Christensen JF Lambrechts A Tsakiris M . The Warburg dance movement library—The WADAMO library: A validation study. Perception. (2019) 48:26–57. doi: 10.1177/0301006618816631, PMID: 30558474

[85] Shafir T Taylor SF Atkinson AP Langenecker SA Zubieta JK . Emotion regulation through execution, observation, and imagery of emotional movements. Brain Cogn. (2013) 82:219–27. doi: 10.1016/j.bandc.2013.03.001, PMID: 23561915 PMC4067484

[86] Shafir T . Movement-based strategies for emotion regulation. In: Handbook on emotion regulation: Processes, cognitive effects and social consequences (2015). p. 231–49. New York: Nova Science.

[87] Shafir T Tsachor RP Welch KB . Emotion regulation through movement: unique sets of movement characteristics are associated with and enhance basic emotions. Front Psychol. (2016) 6:2030. doi: 10.3389/fpsyg.2015.02030, PMID: 26793147 PMC4707271

[88] Tsakiris M Haggard P . Experimenting with the acting self. Cogn Neuropsychol. (2005) 22:387–407. doi: 10.1080/02643290442000158, PMID: 21038257

[89] Marshall AC Gentsch A Schütz-Bosbach S . The interaction between interoceptive and action states within a framework of predictive coding. Front Psychol. (2018) 9:180. doi: 10.3389/FPSYG.2018.00180, PMID: 29515495 PMC5826270

[90] Harduf A Shaked A Yaniv A Salomon R . Disentangling the neural correlates of agency, ownership and multisensory processing. NeuroImage. (2023) 277:120255. doi: 10.1016/j.neuroimage.2023.120255, PMID: 37414232

[91] Tsakiris M . The multisensory basis of the self: From body to identity to others. Q J Exp Psychol. (2017) 70:597–609. doi: 10.1080/17470218.2016.1181768, PMID: 27100132 PMC5214748

[92] Candia-Rivera D Engelen T Babo-Rebelo M Salamone PC . Interoception, network physiology and the emergence of bodily self-awareness. Neurosci Biobehav Rev. (2024) 165:105864. doi: 10.1016/j.neubiorev.2024.105864, PMID: 39208877

[93] Zaidel A Salomon R . Multisensory decisions from self to world. Philos Trans R Soc B. (2024) 378:20220335. doi: 10.1098/rstb.2022.0335, PMID: 37545311 PMC10404927

[94] Engelen T Solcà M Tallon-Baudry C . Interoceptive rhythms in the brain. Nat Neurosci. (2023) 26:1670–84. doi: 10.1038/s41593-023-01425-1, PMID: 37697110

[95] Park HD Bernasconi F Salomon R Tallon-Baudry C Spinelli L Seeck M . Neural sources and underlying mechanisms of neural responses to heartbeats, and their role in bodily self-consciousness: an intracranial EEG study. Cereb Cortex (New York N.Y.: 1991). (2018) 28(7):2351–64. doi: 10.1093/cercor/bhx136, PMID: 28591822

[96] Moccia L Di Luzio M Conte E Modica M Ambrosecchia M Ardizzi M . Sense of agency and its disturbances: A systematic review targeting the intentional binding effect in neuropsychiatric disorders. Psychiatry Clin Neurosci. (2024) 78:3–18. doi: 10.1111/pcn.13601, PMID: 37755315 PMC11488622

[97] Owens AP Allen M Ondobaka S Friston KJ . Interoceptive inference: From computational neuroscience to clinic. Neurosci Biobehav Rev. (2018) 90:174–83. doi: 10.1016/J.NEUBIOREV.2018.04.017, PMID: 29694845

[98] Morie K Crowley M Mayes L Potenza M . The process of emotion identification: Considerations for psychiatric disorders. J Psychiatr Research. (2022) 148:264–74. doi: 10.1016/j.jpsychires.2022.01.053, PMID: 35151218 PMC8969204

[99] Khalsa SS Adolphs R Cameron OG Critchley HD Davenport PW Feinstein JS . Interoception and mental health: A roadmap. Biol Psychiatry: Cogn Neurosci Neuroimaging. (2018) 3:501–13. doi: 10.1016/J.BPSC.2017.12.004, PMID: 29884281 PMC6054486

[100] Friston K . Am i self-conscious? (or does self-organization entail self-consciousness)? Front Psychol. (2018) 9:579. doi: 10.3389/fpsyg.2018.00579, PMID: 29740369 PMC5928749

[101] Corcoran A Pezzulo G Hohwy J . From allostatic agents to counterfactual cognisers: active inference, biological regulation, and the origins of cognition. Biol Philosophy. (2020) 35:32. doi: 10.1007/s10539-020-09746-2

[102] Gallagher S . The past, present and future of time-consciousness: From Husserl to Varela and beyond. Constructivist Foundations. (2017) 13:91–7.

[103] Winnicott DW . Mind and its relation to the psyche-soma. In: Through paediatrics to psycho-analysis. London: Hogarth Press and the Institute of Psycho-Analysis (1954). p. 243–54. 10.1111/j.2044-8341.1954.tb00864.x 13199220

[104] Kyzar E Denfield G . Taking subjectivity seriously: towards a unification of phenomenology, psychiatry, and neuroscience. Mol Psychiatry. (2023) 28:10–6. doi: 10.1038/s41380-022-01891-2, PMID: 36460728 PMC10130907

[105] Sinha P Vogelsang L Vogelsang M Yonas A Diamond S . The temporal scaffolding of sensory organization. Annu Rev Psychol. (2025) 77. doi: 10.1146/annurev-psych-032525-040352, PMID: 41032576 PMC13546142

[106] Arslanova I Kotsaris V Tsakiris M . Perceived time expands and contracts within each heartbeat. Curr Biol. (2023) 33:1389–1395.e4. doi: 10.1016/j.cub.2023.02.034, PMID: 36905931

[107] Ivry RB Spencer RM . The neural representation of time. Curr Opin Neurobiol. (2004) 14:225–32. doi: 10.1016/j.conb.2004.03.013, PMID: 15082329

[108] Hinault T D’Argembeau A Bowler DM La Corte V Desaunay P Provasi J . Time processing in neurological and psychiatric conditions. Neurosci Biobehav Rev. (2023) 154:105430. doi: 10.1016/J.NEUBIOREV.2023.105430, PMID: 37871780

[109] Naghibi N Jahangiri N Khosrowabadi R Eickhoff CR Eickhoff SB Coull JT . Embodying time in the brain: A multi-dimensional neuroimaging meta-analysis of 95 duration processing studies. Neuropsychol Rev. (2023) 34(1):277–98. doi: 10.1007/S11065-023-09588-1, PMID: 36857010 PMC10920454

[110] Marshall AC Gentsch-Ebrahimzadeh A Schütz-Bosbach S . From the inside out: Interoceptive feedback facilitates the integration of visceral signals for efficient sensory processing. NeuroImage. (2022) 251:119011. doi: 10.1016/J.NEUROIMAGE.2022.119011, PMID: 35182753

[111] Candia-Rivera D Catrambone V Thayer JF Gentili C Valenza G . Cardiac sympathetic-vagal activity initiates a functional brain–body response to emotional arousal. Proc Natl Acad Sci United States America. (2022) 119:e2119599119. doi: 10.1073/PNAS.2119599119, PMID: 35588453 PMC9173754

[112] Lake JI LaBar KS Meck WH . Emotional modulation of interval timing and time perception. Neurosci Biobehav Rev. (2016) 64:403–20. doi: 10.1016/J.NEUBIOREV.2016.03.003, PMID: 26972824 PMC5380120

[113] Zhang R Deng H Xiao X . The insular cortex: an interface between sensation, emotion and cognition. Neurosci Bull. (2024) 40:1763–73. doi: 10.1007/s12264-024-01211-4, PMID: 38722464 PMC11607240

[114] Prebble SC Addis DR Tippett LJ . Autobiographical memory and sense of self. psychol Bull. (2013) 139:815–40. doi: 10.1037/A0030146, PMID: 23025923

[115] Menon V . 20 years of the default mode network: A review and synthesis. Neuron. (2023) 111:2469–87. doi: 10.1016/j.neuron.2023.04.023, PMID: 37167968 PMC10524518

[116] Yeshurun Y Nguyen M Hasson U . The default mode network: where the idiosyncratic self meets the shared social world. Nat Rev Neurosci. (2021) 22:3. doi: 10.1038/s41583-020-00420-w, PMID: 33483717 PMC7959111

[117] Raoul L Grosbras M . Relating different dimensions of bodily experiences: Review and proposition of an integrative model relying on phenomenology, predictive brain and neuroscience of the self. Neurosci Biobehav Rev. (2023) 148:105141. doi: 10.1016/j.neubiorev.2023.105141, PMID: 36965863

[118] Koban L Gianaros PJ Kober H Wager TD . The self in context: brain systems linking mental and physical health. Nat Rev Neurosci. (2021) 22:5. doi: 10.1038/s41583-021-00446-8, PMID: 33790441 PMC8447265

[119] Buzsáki G Tingley D . Cognition from the body-brain partnership: exaptation of memory. Annu Rev Neurosci. (2023) 46:191–210. doi: 10.1016/j.tics.2018.07.006, PMID: 36917822 PMC10793243

[120] Allen M Varga S Heck DH . Respiratory rhythms of the predictive mind. psychol Rev. (2022) 130:1066–80. doi: 10.1037/REV0000391, PMID: 35980689

[121] Molnar-Szakacs I Uddin LQ . Anterior insula as a gatekeeper of executive control. Neurosci Biobehav Rev. (2022) 139:104736. doi: 10.1016/J.NEUBIOREV.2022.104736, PMID: 35700753

[122] Barrett L Simmons WK . Interoceptive predictions in the brain. Nat Rev Neurosci. (2015) 16:419–29. doi: 10.1038/nrn3950, PMID: 26016744 PMC4731102

[123] Szpunar KK Spreng RN Schacter DL . A taxonomy of prospection: Introducing an organizational framework for future-oriented cognition. Proc Natl Acad Sci United States America. (2014) 111:18414–21. doi: 10.1073/PNAS.1417144111, PMID: 25416592 PMC4284580

[124] Schiller D Eichenbaum H Buffalo EA Davachi L Foster DJ Leutgeb S . Memory and space: towards an understanding of the cognitive map. J Neurosci. (2015) 35:13904–11. doi: 10.1523/JNEUROSCI.2618-15.2015, PMID: 26468191 PMC6608181

[125] Buzsáki G Tingley D . Space and time: the hippocampus as a sequence generator. Trends Cogn Sci. (2018) 22:853–69. doi: 10.1016/j.tics.2018.07.006, PMID: 30266146 PMC6166479

[126] Buzsáki G McKenzie S Davachi L . Neurophysiology of remembering. Annu Rev Psychol. (2022) 73:187–215. doi: 10.1146/ANNUREV-PSYCH-021721-110002/1, PMID: 34535061 PMC9241447

[127] Casadio C Patané I Ballotta D Candini M Lui F Benuzzi F . Spatial attention modulation of the brain network involved in mental time travel. Neuropsychology. (2023) 38:268–80. doi: 10.1037/NEU0000940, PMID: 38127515

[128] Spelke E . What babies know: Core Knowledge and Composition. London: Oxford University Press (2022).

[129] Thomas A . Cognitive representations of social relationships and their developmental origins. Behavioral Brain Sci. (2024) 29:1–53. doi: 10.31234/osf.io/xhrfu, PMID: 39468838

[130] Suarez-Jimenez B Lazarov A Zhu X Zilcha-Mano S Kim Y Marino CE . Intrusive traumatic re-experiencing domain: functional connectivity feature classification by the ENIGMA PTSD consortium. Biol Psychiatry Global Open Sci. (2024) 4:299–307. doi: 10.1016/J.BPSGOS.2023.05.006, PMID: 38298781 PMC10829610

[131] Dalgleish T Hitchcock C . Transdiagnostic distortions in autobiographical memory recollection. Nat Rev Psychology. (2023) 2:166–82. doi: 10.1038/s44159-023-00148-1

[132] Kearney B Lanius R . Why reliving is not remembering and the unique neurobiological representation of traumatic memory. Nat Ment Health. (2024) 2, 1142–51. doi: 10.1038/s44220-024-00324-z

[133] Lane RD Ryan L Nadel L Greenberg L . Memory reconsolidation, emotional arousal, and the process of change in psychotherapy: New insights from brain science. Behav Brain Sci. (2015) 38:e1. doi: 10.1017/S0140525X14000041, PMID: 24827452

[134] Tomaszewski C Belot RA Essadek A Onumba-Bessonnet H Clesse C . Impact of dance therapy on adults with psychological trauma: a systematic review. Eur J Psychotraumatol. (2023) 14(2):2225152. doi: 10.1080/20008066.2023.2225152, PMID: 37427835 PMC10334851

[135] Shahar-Levy Y . The Function of the human motor system in processes of storing and retrieving preverbal, primal experience. Psychoanalytic Inq. (2001) 21:378–93. doi: 10.1080/07351692109348942

[136] Ianì F . Embodied memories: Reviewing the role of the body in memory processes. Psychonom Bull Rev. (2019) 26:1747–66. doi: 10.3758/S13423-019-01674-X, PMID: 31654375

[137] Van Der Kolk BA . Clinical implications of neuroscience research in PTSD. Ann New York Acad Sci. (2006) 1071:277–93. doi: 10.1196/ANNALS.1364.022, PMID: 16891578

[138] Northoff G Ventura B . Bridging the gap of brain and experience – Converging Neurophenomenology with Spatiotemporal Neuroscience. Neurosci Biobehav Rev. (2025) 173:106139. doi: 10.1016/J.NEUBIOREV.2025.106139, PMID: 40204159

[139] Vaisvaser S King J Orkibi H Aleem H . Neurodynamics of relational aesthetic engagement in creative arts therapies. Rev Gen Psychology. (2024) 28:203–18. doi: 10.1177/10892680241260840

[140] Phelps EA . Human emotion and memory: Interactions of the amygdala and hippocampal complex. Curr Opin Neurobiol. (2004) 14:198–202. doi: 10.1016/j.conb.2004.03.015, PMID: 15082325

[141] de Kloet ER Joëls M . The cortisol switch between vulnerability and resilience. Mol Psychiatry. (2024) 29:20–34. doi: 10.1038/s41380-022-01934-8, PMID: 36599967

[142] Vaisvaser S Lin T Admon R Podlipsky I Greenman Y Stern N . Neural traces of stress: cortisol related sustained enhancement of amygdala-hippocampal functional connectivity. Front Hum Neurosci. (2013) 7:313. doi: 10.3389/fnhum.2013.00313, PMID: 23847492 PMC3701866

[143] Hibel LC Granger DA Blair C Finegood ED Family Life Project Key Investigators . Maternal-child adrenocortical attunement in early childhood: Continuity and change. Dev Psychobiol. (2015) 57:83–95. doi: 10.1002/dev.21266, PMID: 25417896 PMC5317045

[144] Bonini L Rotunno C Arcuri E Gallese V . Mirror neurons 30 years later: implications and applications. Trends Cogn Sci. (2022) 26:767–81. doi: 10.1016/J.TICS.2022.06.003, PMID: 35803832

[145] Keysers C Kaas JH Gazzola V . Somatosensation in social perception. Nat Rev Neurosci. (2010) 11:6. doi: 10.1038/nrn2833, PMID: 20445542

[146] Gallese V . The roots of empathy: The shared manifold hypothesis and the neural basis of intersubjectivity. Psychopathology. (2003) 36:171–80. doi: 10.1159/000072786, PMID: 14504450

[147] Raimo S Boccia M Gaita M Canino S Torchia V Vetere MA . The bodily fundament of empathy: The role of action, nonaction-oriented, and interoceptive body representations. Psychonom Bull Rev. (2023) 30:963–73. doi: 10.3758/S13423-022-02231-9/FIGURES/1, PMID: 36510091

[148] Stern D . Forms of vitality: exploring Dynamic Experience in Psychology, the Arts. London: Oxford University Press (2010).

[149] Di Cesare G Marchi M Lombardi G Gerbella M Sciutti A Rizzolatti G . The middle cingulate cortex and dorso-central insula: A mirror circuit encoding observation and execution of vitality forms. Proc Natl Acad Sci United States America. (2021) 118:e2111358118. doi: 10.1073/PNAS.2111358118/SUPPL_FILE/PNAS.2111358118.SAPP.PDF, PMID: 34716272 PMC8612212

[150] Di Cesare G Koush Y Zeidman P Sciutti A Friston K Rizzolatti G . Bridging feeling and motion: Insula–premotor dynamics in the processing of action vitality forms. Proc Natl Acad Sci. (2025) 122:e2514139122. doi: 10.1073/PNAS.2514139122, PMID: 40928880 PMC12452950

[151] Casartelli L Molteni M Ronconi L . So close yet so far: Motor anomalies impacting on social functioning in autism spectrum disorder. In: Neuroscience and Biobehavioral Reviews, vol. 63. (2016). p. 98–105. doi: 10.1016/j.neubiorev.2016.02.001, PMID: 26855233

[152] Di Cesare G Sparaci L Pelosi A Mazzone L Giovagnoli G Menghini D . Differences in action style recognition in children with autism spectrum disorders. Front Psychol. (2017) 8:1456. doi: 10.3389/FPSYG.2017.01456, PMID: 28928682 PMC5591610

[153] Rochat MJ Gallese V . The blurred vital contours of intersubjectivity in autism spectrum disorder: Early signs and neurophysiological hypotheses. Psychoanalytic Inq. (2022) 42:30–52. doi: 10.1080/07351690.2022.2007022

[154] Daniel S Wimpory D Delafield-Butt JT Malloch S Holck U Geretsegger M . Rhythmic relating: bidirectional support for social timing in autism therapies. Front Psychol. (2022) 13:793258/XML/NLM. doi: 10.3389/FPSYG.2022.793258/XML/NLM 35693509 PMC9186469

[155] Fischman D . Therapeutic relationships and kinesthetic empathy. In: Chaiklin S Wengrower H , editors. The art and science of dance/movement therapy: life is dance. Taylor & Francis Group, Routledge (2009).

[156] Young J . The therapeutic movement relationship in dance/movement therapy: A phenomenological study. Am J Dance Ther. (2017) 39:93–112. doi: 10.1007/S10465-017-9241-9/METRICS

[157] Josef L Goldstein P Mayseless N Ayalon L Shamay-Tsoory SG . The oxytocinergic system mediates synchronized interpersonal movement during dance. Sci Rep. (2019) 9(1):1894. doi: 10.1038/s41598-018-37141-1, PMID: 30760751 PMC6374432

[158] Menon R Neumann ID . Detection, processing and reinforcement of social cues: regulation by the oxytocin system. Nat Rev Neurosci. (2023) 24:761–77. doi: 10.1038/s41583-023-00759-w, PMID: 37891399

[159] Heyes C Catmur C . What happened to mirror neurons? Perspect psychol Sci. (2022) 17:153–68. doi: 10.1177/1745691621990638, PMID: 34241539 PMC8785302

[160] Wang Y Metoki A Xia Y Zang Y He Y Olson IR . A large-scale structural and functional connectome of social mentalizing. NeuroImage. (2021) 236:118115. doi: 10.1016/J.NEUROIMAGE.2021.118115, PMID: 33933599 PMC12317760

[161] Ionta S Heydrich L Lenggenhager B Mouthon M Fornari E Chapuis D . Multisensory mechanisms in temporo-parietal cortex support self-location and first-person perspective. Neuron. (2011) 70:363–74. doi: 10.1016/J.NEURON.2011.03.009, PMID: 21521620

[162] Tsakiris M Costantini M Haggard P . The role of the right temporo-parietal junction in maintaining a coherent sense of one's body. Neuropsychologia. (2008) 46:3014–8. doi: 10.1016/j.neuropsychologia.2008.06.004, PMID: 18601939

[163] Quesque F Brass M . The role of the temporoparietal junction in self-other distinction. Brain Topography. (2019) 32:943–55. doi: 10.1007/s10548-019-00737-5, PMID: 31676934

[164] Blanke O Arzy S . The out-of-body experience: disturbed self-processing at the temporo-parietal junction. Neurosci. (2005) 11:16–24. doi: 10.1177/1073858404270, PMID: 15632275

[165] Alcalá-López D Vogeley K Binkofski F Bzdok D . Building blocks of social cognition: Mirror, mentalize, share? Cortex. (2019) 118:4–18. doi: 10.1016/j.cortex.2018.05.006, PMID: 29903609

[166] Arioli M Cattaneo Z Ricciardi E Canessa N . Overlapping and specific neural correlates for empathizing, affective mentalizing, and cognitive mentalizing: A coordinate-based meta-analytic study. Hum Brain Mapp. (2021) 42:4777–804. doi: 10.1002/HBM.25570, PMID: 34322943 PMC8410528

[167] Ohad T Zvi Y Yeshurun Y . Default mode network synchrony reflects shared understanding. Curr Opin Behav Sci. (2025) 64:101540. doi: 10.1016/J.COBEHA.2025.101540

[168] Sened H Zilcha-Mano S Shamay-Tsoory S . Inter-brain plasticity as a biological mechanism of change in psychotherapy: A review and integrative model. Front Hum Neurosci. (2022) 16:955238. doi: 10.3389/fnhum.2022.955238, PMID: 36092652 PMC9458846

[169] Ramseyer F Tschacher W . Nonverbal synchrony of head- and body-movement in psychotherapy: Different signals have different associations with outcome. Front Psychol. (2014) 5:979/BIBTEX. doi: 10.3389/FPSYG.2014.00979/BIBTEX, PMID: 25249994 PMC4155778

[170] Reinecke KCH Joraschky P Lausberg H . Hand movements that change during psychotherapy and their relation to therapeutic outcome: An analysis of individual and simultaneous movements. Psychother Res. (2022) 32:117–27. doi: 10.1080/10503307.2021.1925989, PMID: 33975526

[171] Schore A . Right brain-to-right brain psychotherapy: recent scientific and clinical advances. Ann Gen Psychiatry. (2022) 21:46. doi: 10.1186/S12991-022-00420-3, PMID: 36403062 PMC9675148

[172] Bolis D Schilbach L . ‘I interact therefore I am’: the self as a historical product of dialectical attunement. Topoi. (2020) 39:521–34. doi: 10.1007/S11245-018-9574-0

[173] Fini C Bardi L Bolis D Fusaro M Lisi MP Michalland AH . The social roots of self development: from a bodily to an intellectual interpersonal dialogue. psychol Res. (2023) 87:1683–95. doi: 10.1007/S00426-022-01785-6, PMID: 36595049

[174] Payne H . Relational integrative psychotherapy and the discipline of authentic movement. Am J Dance Ther. (2024) 46:34–51. doi: 10.1007/S10465-023-09394-5

[175] Adler J . Offering from the Conscious Body: The Discipline of Authentic Movement. London: Simon & Schuster (2002).

[176] Marcow-Speiser V Franklin M . Authentic movement as a meditative practice. Journal of Pedagogy, Pluralism, and Practice. (2007) 3(4):68.

[177] Barkai Y . On the authentic movement model: A space for creation—A place to be. Am J Dance Ther. (2022) 44:4–20. doi: 10.1007/S10465-022-09354-5

[178] Hasson U Frith CD . Mirroring and beyond: coupled dynamics as a generalized framework for modelling social interactions. Philos Trans R Soc B: Biol Sci. (2016) 371:20150366. doi: 10.1098/RSTB.2015.0366, PMID: 27069044 PMC4843605

[179] Shamay-Tsoory SG Saporta N Marton-Alper IZ Gvirts HZ . Herding brains: A core neural mechanism for social alignment. Trends Cogn Sci. (2019) 23:174–86. doi: 10.1016/J.TICS.2019.01.002, PMID: 30679099

[180] Lotter LD Kohl SH Gerloff C Bell L Niephaus A Kruppa JA . Revealing the neurobiology underlying interpersonal neural synchronization with multimodal data fusion. Neurosci Biobehav Rev. (2023) 146:105042. doi: 10.1016/J.NEUBIOREV.2023.105042, PMID: 36641012

[181] Hu Y Cheng X Pan Y Hu Y . The intrapersonal and interpersonal consequences of interpersonal synchrony. Acta Psychol. (2022) 224:103513. doi: 10.1016/J.ACTPSY.2022.103513, PMID: 35093851

[182] Koole SL Tschacher W . Synchrony in psychotherapy: A review and an integrative framework for the therapeutic alliance. Front Psychol. (2016) 7:862. doi: 10.3389/FPSYG.2016.00862, PMID: 27378968 PMC4907088

[183] Zhang Y Meng T Yang Y Hu Y . Experience-dependent counselor-client brain synchronization during psychological counseling. ENeuro. (2020) 7:1–10. doi: 10.1523/ENEURO.0236-20.2020, PMID: 32878962 PMC7519169

[184] Ellingsen DM Isenburg K Jung C Lee J Gerber J Mawla I . Dynamic brain-to-brain concordance and behavioral mirroring as a mechanism of the patient-clinician interaction. Sci Adv. (2020) 6:eabc1304. doi: 10.1126/sciadv.abc1304, PMID: 33087365 PMC7577722

[185] Feldman R . What is resilience: an affiliative neuroscience approach. World Psychiatry. (2020) 19:132–50. doi: 10.1002/wps.20729, PMID: 32394561 PMC7215067

[186] Zilcha-Mano S Goldstein P Dolev-Amit T Shamay-Tsoory S . Oxytocin synchrony between patients and therapists as a mechanism underlying effective psychotherapy for depression. J Consult Clin Psychol. (2021) 89:49. doi: 10.1037/ccp0000619, PMID: 33507775

[187] Tschacher W Meier D . Physiological synchrony in psychotherapy sessions. Psychother Res. (2020) 30:558–73. doi: 10.1080/10503307.2019.1612114, PMID: 31060474

[188] Marci CD Ham J Moran E Orr SP . Physiologic correlates of perceived therapist empathy and social-emotional process during psychotherapy. J Nervous Ment Dis. (2007) 195:103–11. doi: 10.1097/01.NMD.0000253731.71025.FC, PMID: 17299296

[189] Mu Y Guo C Han S . Oxytocin enhances inter-brain synchrony during social coordination in male adults. Soc Cogn Affect Neurosci. (2016) 11:1882–93. doi: 10.1093/SCAN/NSW106, PMID: 27510498 PMC5141958

[190] Lucherini Angeletti L Ventura B Galassi F Castellini G Ricca V Scalabrini A . The self and its intersubjective synchrony in psychotherapy: A systematic review. Clin Psychol Psychother. (2025) 32:e70110. doi: 10.1002/CPP.70110, PMID: 40590497 PMC12211550

[191] Gallotti M Fairhurst M Frith C . Alignment in social interactions. Consciousness Cogn. (2017) 48:253–61. doi: 10.1016/j.concog.2016.12.002, PMID: 28033550

[192] Rosso M Heggli O Maes P Vuust P Leman M . Mutual beta power modulation in dyadic entrainment. Neuroimage. (2022) 257:119326. doi: 10.1016/j.neuroimage.2022.119326, PMID: 35667334

[193] Friston K Frith C . A duet for one. Consciousness Cogn. (2015) 36:390–405. doi: 10.1016/j.concog.2014.12.003, PMID: 25563935 PMC4553904

[194] Mayo O Shamay-Tsoory S . Dynamic mutual predictions during social learning: A computational and interbrain model. Neurosci Biobehav Rev. (2024) 157:105513. doi: 10.1016/J.NEUBIOREV.2023.105513, PMID: 38135267

[195] Chen W Liu T Dong D . Sharing vitality at the moments of meeting. J Consciousness Stud. (2024) 31:60–84. doi: 10.53765/20512201.31.11.060

[196] Bolis D Dumas G Schilbach L . Interpersonal attunement in social interactions: from collective psychophysiology to inter-personalized psychiatry and beyond. Philos Trans R Soc B. (2023) 378:20210365. doi: 10.1098/RSTB.2021.0365, PMID: 36571122 PMC9791489

[197] Konrad K Gerloff C Kohl SH Mehler DMA Mehlem L Volbert EL . Interpersonal neural synchrony and mental disorders: unlocking potential pathways for clinical interventions. Front Neurosci. (2024) 18:1286130. doi: 10.3389/FNINS.2024.1286130, PMID: 38529267 PMC10962391

[198] Schilbach L Redcay E . Synchrony across brains. Annu Rev Psychol. (2025) 76:883–911. doi: 10.1146/annurev-psych-080123-101149, PMID: 39441884 PMC12668431

[199] Bentzen M . Dances of connection: Neuroaffective development in clinical work with attachment. Body Movement Dance Psychother. (2015) 10:211–26. doi: 10.1080/17432979.2015.1064479

[200] Atzil-Slonim D Soma CS Zhang X Paz A Imel ZE . Facilitating dyadic synchrony in psychotherapy sessions: systematic review and meta-analysis. Psychother Res. (2023) 33:898–917. doi: 10.1080/10503307.2023.2191803, PMID: 37001119

[201] Zilcha-Mano S . How getting in sync is curative: Insights gained from research in psychotherapy. psychol Rev. (2024) 33132(2):470–87. doi: 10.1037/rev0000471, PMID: 38358717

[202] Biondo J . Dance/movement therapy as a holistic approach to diminish health discrepancies and promote wellness for people with schizophrenia: a review of the literature. F1000Research. (2023) 12:33. doi: 10.12688/F1000RESEARCH.127377.2, PMID: 37593363 PMC10429376

[203] Vaisvaser S . Moving along and beyond the spectrum: Creative group therapy for children with autism. Front Psychol. (2019) 10:417. doi: 10.3389/fpsyg.2019.00417, PMID: 30914987 PMC6423063

[204] Xu M Morimoto S Hoshino E Suzuki K Minagawa Y . Two-in-one system and behavior-specific brain synchrony during goal-free cooperative creation: an analytical approach combining automated behavioral classification and the eventrelated generalized linear model. Neurophotonics. (2023) 10:13511. doi: 10.1117/1.NPh.10.1.013511, PMID: 36789283 PMC9917717

[205] Grasso-Cladera A Costa-Cordella S Mattoli-Sánchez J Vilina E Santander V Hiltner SE . Embodied hyperscanning for studying social interaction: A scoping review of simultaneous brain and body measurements. Soc Neurosci. (2025) 20(3):163–79. doi: 10.1080/17470919.2024.2409758, PMID: 39387663

[206] Barnstaple R Protzak J DeSouza J Gramann K . Mobile brain/body Imaging in dance: A dynamic transdisciplinary field for applied research. Eur J Neuroscience. (2021) 54:8355–63. doi: 10.1111/ejn.14866, PMID: 32544262

[207] Kaiser N Avendano-Diaz JC . The ConNECT approach: toward a comprehensive understanding of meaningful interpersonal moments in psychotherapy and beyond. Front Humen Neurosci. (2025) 19:1549203. doi: 10.3389/FNHUM.2025.1549203, PMID: 40177164 PMC11961974

[208] Poikonen H Duberg A Eriksson M Eriksson-Crommert M Lund M Möller M . InMotion”—Mixed physical exercise program with creative movement as an intervention for adults with schizophrenia: study protocol for a randomized controlled trial. Front Hum Neurosci. (2023) 17:1192729. doi: 10.3389/FNHUM.2023.1192729, PMID: 37476005 PMC10354340

[209] Moffat R Casale CE Cross ES . Mobile fNIRS for exploring inter-brain synchrony across generations and time. Front Neuroergonomics. (2024) 4:1260738. doi: 10.3389/fnrgo.2023.1260738, PMID: 38234472 PMC10790948

[210] Sened H Gorst Kaduri K Nathan Gamliel H Rafaeli E Zilcha-Mano S Shamay-Tsoory S . Inter-brain plasticity as a mechanism of change in psychotherapy: A proof of concept focusing on test anxiety. Psychother Res. (2025) 20:1–15. doi: 10.1080/10503307.2025.2451798, PMID: 39832304

